# Perinatal Outcomes Related to the Presence of a Nuchal Cord During Delivery: A Retrospective Cohort Study

**DOI:** 10.3390/diagnostics15101197

**Published:** 2025-05-09

**Authors:** Gabriel Viana Silva, Carolina Toledo Gontijo, Ana Paola Cruz Lunguinho, Mário Sérgio Gomes Caetano, Gustavo Yano Callado, Edward Araujo Júnior, Alberto Borges Peixoto

**Affiliations:** 1 Department of Obstetrics and Gynecology, Federal University of Triângulo Mineiro (UFTM), Uberaba 38025-440, MG, Brazil; gabrielvianasilva1@gmail.com (G.V.S.); carol.toledo1993@gmail.com (C.T.G.); apclunguinho@hotmail.com (A.P.C.L.); mscaetano73@gmail.com (M.S.G.C.); 2 Albert Einstein Israelite College of Health Sciences, Albert Einstein Israelite Hospital, São Paulo 05653-120, SP, Brazil; gycallado@gmail.com; 3 Department of Obstetrics, Paulista School of Medicine—Federal University of São Paulo (EPM-UNIFESP), São Paulo 04023-062, SP, Brazil; araujojred@terra.com.br; 4 Discipline of Woman Health, Municipal University of São Caetano do Sul (USCS), São Caetano do Sul 09521-160, SP, Brazil; 5 Gynecology and Obstetrics Service, Mário Palmério University Hospital, University of Uberaba (UNIUBE), Uberaba 38050-175, MG, Brazil

**Keywords:** nuchal cord, tight cord, delivery, adverse perinatal outcomes

## Abstract

**Objective**: To evaluate and compare whether the presence of a nuchal cord (NC) and its characteristics had a negative impact on perinatal outcomes during delivery. **Methods**: This was a retrospective cohort study that analyzed the medical records of pregnant women from March 2020 to June 2023. Pregnant women were divided into groups with and without an NC. Singleton pregnancies ≥ 37 weeks were included, excluding fetal malformations, chromosomal anomalies, and cases with missing data and cord blood gas. **Results**: Of the 3364 medical records analyzed, 466 were included—366 without and 100 with an NC. Among the cases with an NC, 91% had one loop and 9% had ≥ two loops; 82% were loose and 18% were tight. Pregnant women with an NC had a higher gestational age (39.7 vs. 39.1 weeks, *p* = 0.006), fewer deliveries (1.0 vs. 2.0, *p* = 0.035), and a higher prevalence of cesarean sections (99% vs. 60.4%, *p* < 0.001). An NC was associated with a lower Apgar score at the 1st minute (8 vs. 9, *p* = 0.014) and higher arterial cord blood pH (7.27 vs. 7.24, *p* = 0.020). The presence of a tight cord was significantly associated with a 7.52-fold increased risk of an Apgar score < 7 at the 1st minute [x^2^(1) = 5.92, OR: 7.52, 95% CI: 1.51–37.31, R^2^ Nagelkerke: 0.14, *p* = 0.014]. **Conclusions**: There was no effect of the presence of an NC on adverse perinatal outcomes. However, the presence of a tight NC was associated with an increased risk of an Apgar score < 7 at the 1st minute, but no other effect on neonatal outcomes.

## 1. Introduction

In 2015, approximately 30,000 women and adolescents died as a result of complications related to pregnancy and childbirth [1]. In 2016, the World Health Organization (WHO) published a document on antenatal care in order to achieve more positive perinatal and maternal outcomes. To this end, recommendations were made for the prevention and early detection of specific conditions related to pregnancy. A secondary analysis of the WHO antenatal care guidelines suggested that the increase in perinatal mortality was likely due to an increase in the number of stillbirths [2]. Therefore, quality medical care during the perinatal period can provide better maternal–fetal outcomes and thereby reduce morbidity and mortality in this group.

Prevention and identification of possible risk factors for complications during pregnancy and childbirth are fundamental to prenatal care. Some risk factors, such as hypertensive disorders of pregnancy, are well described and, with proper care, can have outcomes similar to a low-risk pregnancy, with positive outcomes in the perinatal period. However, there are some factors identified during prenatal care or even during delivery that are not yet well described regarding whether or not they negatively influence perinatal outcomes. Among these, the presence of a nuchal cord (NC), which can be seen on ultrasound scans performed during prenatal care or during childbirth, has not yet been well defined regarding whether or not it acts as a worse prognostic factor in perinatal outcomes [3,4].

The term ‘nuchal cord’ refers to the umbilical cord wrapped 360° around the fetal neck [5]. There may be one 360° turn or more than one, characterizing two or more cord loops. It is classified as either a loose NC that can be manually disengaged before the fetal head is detached during delivery, or a tight NC if it cannot be manually disengaged, requiring the umbilical cord to be clamped and cut [6]. The presence of an NC may be noted during prenatal ultrasound or even during delivery. The prevalence of a tight NC is observed in 6.6% of live births, while a loose NC is observed in 21.6% [7]. Therefore, it is necessary to determine whether or not the presence of an NC adversely affects perinatal outcomes to guide clinical decision-making and prevent possible complications.

Although the presence of an NC is frequently observed during delivery, the establishment of a clear cause-and-effect relationship between the cord around the fetal neck and adverse perinatal outcomes remains a subject of debate. Studies suggest that an NC is not significantly associated with increased perinatal mortality—with similar outcomes observed in births with and without an NC [8]. However, studies report associations between a tight NC and complications such as non-reassuring fetal heart rate patterns, increased rates of operative delivery, and neonatal intensive care unit (NICU) admissions [7]. Nevertheless, association does not imply causation. Confounding variables, including labor duration, fetal positioning, and obstetric interventions, may influence these outcomes. Additionally, no significant link has been found between NC presence and long-term cardiovascular or respiratory morbidity in offspring [8]. However, an NC can be associated with adverse outcomes during delivery, including higher rates of emergency cesarean sections, preterm births, and consequently, increased risk of intraventricular hemorrhage and retinopathy of prematurity [7].

Multiple NC loops, particularly three or more, have been linked to more severe complications such as intrauterine fetal death, growth restriction, and low Apgar scores [8,9]. While an NC can cause abnormalities in umbilical cord gases [10,11] and lead to complications during labor [4], most fetuses can compensate for transient reductions in umbilical blood flow, resulting in successful deliveries [12]. Additionally, multiple umbilical cord loops, compared with single loops or no loops, are associated with an increased risk of adverse neonatal outcomes, such as low Apgar scores and fetal acidosis [13].

The aim of this study was to evaluate whether the presence and characteristics of an NC negatively impact perinatal outcomes at the time of delivery.

## 2. Methods

### 2.1. Study Design and Setting

This was a retrospective cohort study carried out at the Clinics Hospital of the Federal University of Triângulo Mineiro (UFTM), Uberaba, Brazil, through the evaluation of medical records of pregnant women during labor assistance, from March 2020 to March 2023. The participants were divided into two groups according to the presence or absence of a nuchal cord.

### 2.2. Inclusion and Exclusion Criteria

Inclusion criteria were singleton pregnancies with a gestational age ≥ 37 weeks undergoing vaginal, instrumental, or cesarean section. The gestational age was confirmed by a first-trimester ultrasound. Exclusion criteria were (1) fetal malformations and chromosomal abnormalities diagnosed by ultrasound or clinically after delivery; (2) the absence of documentation on the presence or absence of an NC at the time of delivery; and (3) the absence of umbilical cord blood gas analysis in the medical records, which precluded the evaluation of acid–base status regardless of NC status.

### 2.3. Definitions

An NC was considered to be the presence of at least one loop of cord wrapped 360° around the fetal neck at the time of cephalic pole extraction by vaginal delivery or cesarean section. Multiple NCs were defined as the presence of two or more loops. A tight NC was defined as one that could not be manually disengaged before the fetal head was delivered, requiring the umbilical cord to be clamped and cut. The institutional protocol does not include routine ultrasound screening for an NC at any time during prenatal care. In addition, no specific management is planned if an NC is diagnosed before delivery. Epidural analgesia for pain relief or cesarean section is indicated whenever clinically necessary.

### 2.4. Umbilical Cord Blood Gas Collection and Analysis

In our service, umbilical cord blood gases are routinely performed immediately after vaginal, instrumental, or cesarean section. After fetal expulsion, the obstetrician clamps a section of the umbilical cord approximately 20 cm in length, from which 1 mL of arterial blood, followed by 1 mL of venous blood from the umbilical cord, is collected in a 3 mL blood gas syringe containing heparin (BD Luer Lok–New Jersey, United States). The blood gas syringes are transported to the clinical analysis laboratory within a maximum of 30 min after collection. The parameters analyzed in the umbilical cord blood gas were arterial pH and venous pH. An umbilical arterial pH < 7.1 and/or an umbilical venous pH < 7.2 were considered a practical threshold to define pathological fetal acidemia. If there was a pH difference < 0.02, it was considered that blood had been collected from the same vessel or that a mixture of arterial and venous blood had occurred [9,10,14,15,16].

### 2.5. Variables Collected

The following variables were assessed: age, ethnicity, body mass index (BMI), number of pregnancies, number of previous deliveries, pre-existing medical conditions (high-risk pregnancy), gestational age at delivery, type of delivery, birthweight, duration of labor, Apgar score at the 1st minute, Apgar score at the 5th minute, arterial cord blood pH, venous cord blood pH, presence of an NC, number of loops, and the presence of a loose or tight NC.

### 2.6. Definition of Adverse Perinatal Outcomes

The following were considered adverse perinatal outcomes: Apgar score < 7 at the 1st minute, Apgar score < 7 at the 5th minute, meconium-stained amniotic fluid, fetal acidemia at birth, need for oxygen therapy immediately after birth, neonatal intensive care unit admission, neonatal death within 48 h, and fetal death at delivery. The presence of at least one adverse perinatal outcome was considered a composite adverse outcome.

### 2.7. Sample Size Calculation

G*power 3.1 was used to calculate sample sizes. A total of 1143 study participants were required to determine the association between NC and perinatal adverse outcomes, considering an effect size of 0.30, a type I error probability (α) of 0.05, and a power of 0.80. To determine the effect of the NC on continuous variables, 278 participants were required, considering an effect size of 0.30, α = 0.05, and a power of 0.80. To analyze the correlation between continuous variables, 106 participants were required, assuming an effect size of 0.50, α = 0.05, and a power of 0.80. To evaluate predictors of adverse perinatal outcomes, 242 participants were required, assuming an odds ratio (OR) of 1.5, α = 0.05, and a power of 0.80.

### 2.8. Ethical Approval

Our investigations were carried out following the rules of the Declaration of Helsinki of 1975, revised in 2013. The study was approved by the Ethics Committee of UFTM (CAAE: 68241823.4.0000.8667).

### 2.9. Statistical Analysis

Data were transferred to an Excel 2019 spreadsheet (Microsoft Corp., Redmond, WA, USA) and analyzed using SPSS version 20.0 (SPSS Inc., Chicago, IL, USA) and Prism GraphPad version 7.0 (GraphPad Software, San Diego, CA, USA). Quantitative variables were first subjected to the D’Agostino–Pearson normality test. Variables with a normal distribution were presented as means and standard deviations (SD), while those with a non-normal distribution were presented as medians and interquartile ranges (IQR). Categorical variables were described using absolute and relative frequencies, and were presented in tables. The Chi-squared test was used to analyze differences between categorical variables. The Mann–Whitney test was used for differences between continuous variables. Spearman’s test was used to analyze correlations between continuous variables with a non-parametric distribution. Binary logistic regression was initially used to calculate the OR for adverse perinatal outcomes. Stepwise binary logistic regression was then used to identify the best predictors of adverse perinatal outcomes. The level of significance for all tests was set at α = 0.05.

## 3. Results

**Figure 1:**
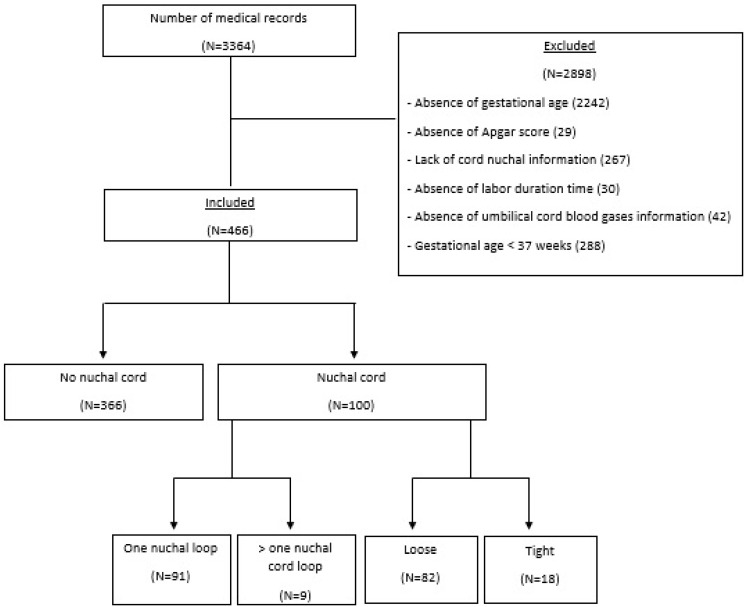
Flowchart of included cases.

From March 2020 to June 2023, 3364 pregnant women who underwent vaginal, instrumental, and cesarean delivery were retrospectively selected through the analysis of medical records. A total of 2242 cases were excluded due to a lack of information on gestational age at delivery, 29 cases were excluded due to a lack information on of Apgar score, 267 cases were excluded due to missing information on the presence of NC, 30 cases were excluded due to a lack of information on the duration of labor, 42 cases were excluded due to a lack of information on umbilical cord blood gases and 288 cases due were excluded due to a gestational age < 37 weeks. A total of 466 cases were included in the final statistical analysis. Among the included cases, 366 (78.5%) had no NC at delivery and 100 (21.5%) had an NC. Among the cases with an NC, 91 (91.0%) had only one cord loop and 9 (9.0%) had more than one loop. Among all the cases, 82 (82.0%) had a loose NC and 18 (18.0%) had a tight NC (Figure 1).

Pregnant women with an NC at delivery had fewer previous deliveries (1.0 vs. 2.0, *p* = 0.035), a higher gestational age at delivery (39.7 vs. 39.1 weeks, *p* = 0.006), a lower Apgar score at the 1st minute (8.0 vs. 9.0, *p* = 0.014), and a higher arterial cord blood pH (7.27 vs. 7.24, *p* = 0.020). The presence of an NC had no effect on maternal age (*p* = 0.814), BMI (*p* = 0.277), the number of deliveries (*p* = 0.167), birthweight (*p* = 0.206), duration of labor (*p* = 0.193), Apgar score at the 5th minute (*p* = 0.636), or venous cord blood pH (*p* = 0.191). The presence of an NC was significantly associated with the mode of delivery (*p* < 0.001). Participants with an NC at delivery had a higher prevalence of vaginal delivery than those without (99.0% vs. 60.4%, *p* < 0.001). There was no significant association between an NC and high-risk pregnancy (*p* = 0.347) or ethnicity (*p* = 0.376) (Table 1).

The presence of an NC was not a significant predictor of an Apgar score < 7 at the 1st minute (*p* = 0.413), an Apgar score < 7 at the 5th minute (*p* = 0.993), meconium at birth (*p* = 0.059), acidemia at birth (*p* = 0. 262), oxygen therapy (*p* = 0.697), neonatal intensive care unit admission (*p* = 0.789), neonatal death within 48 h (*p* = 0.601), or composite adverse outcomes (*p* = 0.500). There were no cases of fetal death at delivery (Table 2).

Participants with a tight NC at delivery had a higher BMI (32.9 vs. 28.8 Kg/m^2^, *p* = 0.045), fewer previous deliveries (1.0 vs. 2.0, *p* = 0.022), a higher gestational age at delivery (39.9 vs. 39.7 weeks, *p* = 0.003), a longer duration of labor (40.5 vs. 30.0 min, *p* < 0.001), a lower Apgar score at the 1st minute (8.0 vs. 9.0, *p* = 0.007), and a lower arterial cord blood pH (7.23 vs. 7.28, *p* = 0.025). There was no significant effect of a tight NC on maternal age (*p* = 0.828), the number of deliveries (*p* = 0.165), the number of cord loops (*p* = 0.357), birthweight (*p* = 0.082), the Apgar score at the 5th minute (*p* = 0.726), venous cord blood pH (*p* = 0.099), ethnicity (*p* = 0.576), high-risk pregnancy (*p* = 0.410), or the mode of delivery (*p* = 0.638) (Table 3).

**Figure 2:**
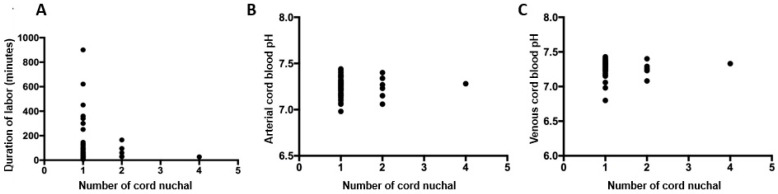
Correlation between number of nuchal cord loops and duration of labor (**A**), arterial cord blood pH (**B**), and venous cord blood pH (**C**). Spearman’s correlation test. *p* < 0.05.

There was no significant correlation between the number of cord loops and the duration of labor (r = −0.008, *p* = 0.935), arterial cord blood pH (r = −0.030, *p* = 0.747), or venous cord blood pH (r = −0.132, *p* = 0.208) (Figure 2).

The presence of a tight NC was a significant predictor of an Apgar score < 7 at the 1st minute (*p* = 0.014). It was significantly associated with a 7.52-fold increased risk of an Apgar score < 7 at the 1st minute [X^2^(1) = 5.92, OR: 7.52, 95% CI: 1.51–37.31, R^2^ Nagelkerke: 0.14, *p* = 0.014]. However, the presence of a tight NC was not a significant predictor of an Apgar score < 7 at the 5th minute (*p* = 0.278), meconium at birth (*p* = 0.898), acidemia at birth (*p* = 0.712), oxygen therapy (*p* = 0.065), neonatal intensive care unit admission (*p* = 0.503), neonatal death within 48 h (*p* = 0.601), or composite adverse outcomes (*p* = 0.163). There were no cases of neonatal death within 48 h or fetal death at delivery in participants with a loose or tight NC during delivery (Table 4).

A stepwise binary logistic regression model was constructed to assess whether the presence of an NC, a tight NC, the number of cord loops, the duration of labor, and the gestational age at delivery were predictors of an Apgar score < 7 at the 1st minute. A tight NC was found to be an independent predictor of an Apgar score < 7 at the 1st minute [X^2^(1) = 5.92 OR: 7.52, 95% CI: 1.51–37.31, R^2^ Nagelkerke: 0.14, *p* = 0.014]. The presence of an NC (*p* = 0.415), the number of cord loops (*p* = 0.311), the duration of labor (*p* = 0.311), and the gestational age at delivery (*p* = 0.494) were not predictors of an Apgar score < 7 at the 1st minute.

## 4. Discussion

The presence of a tight NC at delivery is significantly associated with an increased risk of an Apgar score < 7 at the 1st minute. An Apgar score < 7 at the 1st minute is generally associated with intrapartum asphyxia and, when persisting at the 5th minute, with neonatal neurological sequelae. This finding is consistent with the literature, which suggests umbilical cord compression as a cause of temporary decreased fetal oxygenation, resulting in lower Apgar scores immediately after birth [11,17].

Pergialiotis et al. [18], in their systematic review, demonstrated a change in clinical outcome in the presence of an NC, highlighting the need for early ultrasound diagnosis aimed at planning appropriate perinatal management to minimize adverse outcomes. Zahedi-Spung et al. [18] analyzed 8580 cases and found an increased prevalence of an Apgar score < 7 at the 1st minute, associated with hypercapnia on cord blood gases in fetuses with an NC. The authors concluded that, despite these adverse outcomes, the presence of an NC did not alter perinatal morbidity and mortality. In our study, even in the absence of antenatal ultrasound screening, we did not observe an increased risk of adverse perinatal outcomes associated with the presence of an NC at delivery.

In our study, a predominance of vaginal deliveries was observed even in the presence of an NC. The presence of a tight NC during labor is associated with an increased need for obstetric interventions, including emergency cesarean sections and operative vaginal deliveries. An NC may lead to non-reassuring fetal heart rate patterns, often resulting in interventions, such as an emergency cesarean section [4,14]. Additionally, the presence of multiple NC loops is related to a higher incidence of operative vaginal deliveries, such as those using forceps or vacuum extraction [9,19]. Młodawska et al. [20] reported an increased prevalence of cesarean sections in the presence of an NC; however, similarly to our findings, they observed no relevant clinical or laboratory differences between the groups. We believe that the lack of antenatal screening for an umbilical NC in our service did not influence decisions about the mode of delivery, and thus contributed to the high prevalence of vaginal deliveries among participants with an NC.

The effect of an NC on maternal and neonatal outcomes has been previously studied, but conclusions remain inconsistent. Maternal age does not seem to be significantly associated with the presence of an NC, although one study reported a slightly higher maternal BMI in cases involving vacuum-assisted deliveries [21]. Regarding parity, NCs have been linked to lower rates of nulliparity, suggesting that women with an NC may have had more previous deliveries [21]. Some studies have also associated NCs with lower birthweights, although this finding is not universally consistent across all research [21,22,23]. Hypotheses for these associations include the possibility that heavier or more multiparous women may experience altered intrauterine dynamics, potentially facilitating cord entanglement, and that lower birthweights might reflect subtle chronic hypoxia caused by intermittent cord compression. However, the absence of consistent findings suggests that NC formation is likely a random event related more to fetal mobility and cord length than to maternal characteristics or fetal growth.

In terms of labor characteristics, the presence of an NC has been associated with a shorter second stage of labor during vacuum-assisted deliveries, although not all studies corroborate this result. An NC in vaginal deliveries at term has been associated with an increased rate of operative vaginal and cesarean delivery in nulliparous women [24]. Additionally, concerning neonatal outcomes, the presence of an NC may be associated with slightly lower Apgar scores at 1 and 5 min [24]. Nevertheless, other investigations have not demonstrated significant differences in 5 min Apgar scores or venous cord blood pH [25], suggesting that the majority of NCs do not result in sustained hypoxic injury [21,26]. Consistent with these latter findings, our analysis showed no significant association between the presence of an NC and maternal age, BMI, parity, birthweight, the duration of labor, the Apgar score at the 5th minute, or venous cord blood pH, reinforcing the hypothesis that an NC, in most cases, represents a benign finding without major clinical repercussions when appropriately managed during labor and delivery.

Schäffer et al. [22] conducted a retrospective cohort study involving 11,748 pregnant women with planned vaginal deliveries, including 9574 term and 2174 post-term deliveries. The incidence of an NC in term and post-term deliveries was 33.7% and 35.1%, respectively. Multiple NC loops were present in 5.8% of term and 5.5% of post-term deliveries. Meconium at birth was significantly increased only in cases of multiple NC loops in post-term deliveries. Unfavorable neonatal blood gas parameters were significantly more frequent in all NC groups. Apgar scores < 7 at the 5th minute and neonatal intensive care unit admission did not differ significantly between groups. Compared to our study, which included only term pregnancies, we observed a lower rate of NCs (33.7% vs. 21.5%) and a higher rate of multiple cord loops (5.8% vs. 9.0%).

In our study, we observed a higher arterial pH in cord blood among participants with a single loop and lower arterial pH among those with a tight loop. Despite these differences, no clinical relevance was identified, as there was no difference in the presence of acidemia at birth among the studied groups. Rhoades et al. [27] performed a population-based case–control study including 3000 newborns with an NC and 3000 without. An NC was associated with increased risks of fetal distress (OR 2.7, 95% CI 2.1–3.4), meconium at birth (OR 2.1, 95% CI 1.7–2.6), an Apgar score < 7 at the 5th minute (OR 1.6, 95% CI 1.1–2.4) and assisted ventilation < 30 min. In our study, however, the presence of an NC was not associated with adverse perinatal outcomes.

In our study, we did not evaluate other umbilical cord variables such as the umbilical cord coiling index (UCI). The current medical literature does not establish a clear association between umbilical cord coiling and fetal acidemia at birth. Atalla et al. [28] investigated the relationship between the morphological characteristics of the umbilical cord and the acid–base status of newborns and found correlations between umbilical venous pH and characteristics such as cord length and the number of vascular coils, but did not identify a direct relationship between cord coiling indexes and fetal acidemia. In a meta-analysis, Pergialiotis et al. [29] evaluated the UCI and its association with adverse pregnancy outcomes, including fetal acidemia. Although hypercoiled cords have been associated with fetal acidemia, the analysis suggests that further studies are needed to determine whether antenatal assessment of the UCI can be used routinely in clinical practice. The variability in the results regarding the presence of umbilical cord coiling and its morphology may be attributed to additional factors such as placental morphology and placental blood flow, which affect maternal–fetal gas exchange [23].

Vasa et al. [30] assessed 2530 pregnant women, reporting an incidence of an NC of 23.5% and of a tight NC of 1.9%. Among newborns with an NC, 4.2% required resuscitation, and 3.2% were admitted to the neonatal intensive care unit. The frequency of an NC increased from 15.6% at ≤36 weeks to 22.8% at ≥37 weeks. Elevated arterial and venous blood cord pH were observed in NC cases. Compared to our study, we observed similar rates of NCs in general and lower rates of tight NCs. We also observed elevated arterial blood cord pH values in cases with an NC.

The current medical literature provides evidence for the association between tight cord entanglement and low Apgar scores at the 1st minute. Zabit et al. [31] demonstrated that umbilical cord entanglement is significantly associated with Apgar scores < 7 at the 1st minute, with an OR of 1.21. This finding is supported by the meta-analysis by Pergialiotis et al. [29], who found an increased risk of low Apgar scores at the 1st minute in cases of cord entanglement, with a relative risk (RR) of 1.75. In addition, Schreiber et al. [9] reported that multiple NC entanglements, especially three loops, are associated with a higher incidence of Apgar scores < 7 at the 1st minute. These studies suggest that tight cord ties may lead to transient cord compression, resulting in temporary fetal hypoxia, which may negatively impact the initial Apgar score.

In our study, a lower Apgar score at the 1st minute and higher arterial blood cord pH were observed in cases with an NC, but there was no significant difference in the rates of an Apgar score < 7 at the 1st minute or acidemia at birth. On the other hand, we observed that the presence of a tight nuchal cord was an independent predictor of an Apgar score < 7 at the 1st minute. The presence of a tight NC was also associated with a longer labor duration and a 7.52-fold increased risk of an Apgar score < 7 at the 1st minute. Although a tight NC can lead to a reduction in the Apgar score at the 1st minute, there was no increase in the risk of acidemia at birth, oxygen therapy, neonatal intensive care unit admission, or neonatal death within the first 48 h. This suggests that rapid obstetric intervention was effective in preventing severe complications at birth [7].

One of the limitations of our study was the retrospective nature of the data, which led to the exclusion of a significant number of cases due to the absence of key data at the time of hospitalization and their lack of documentation in the electronic medical records. Unfortunately, we did not have access to other risk factors for perinatal asphyxia, such as the method of labor induction or socioeconomic status, which can influence access to prenatal care, maternal nutrition, and overall pregnancy monitoring, thereby impacting perinatal outcomes. Nevertheless, it is worth noting that the number of evaluated pregnant women exceeded the expected total of 176, reaching a total of 466, thus justifying the routine collection of cord blood gases in our service.

## 5. Conclusions

There was no effect of the presence of an NC on adverse perinatal outcomes. The presence of a tight NC during delivery was significantly associated with an increased risk of an Apgar score < 7 at the 1st minute, but was not associated with oxygen therapy, acidemia at birth, an Apgar score < 7 at the 5th minute, neonatal intensive care unit admission, or neonatal death within 48 h. Finally, the presence of an NC had no significant clinical or laboratory relevance for fetal morbidity and mortality, and efficient obstetric management was associated with the significant minimization of perinatal risks.

## Figures and Tables

**Table 1:** The presence of a nuchal cord according to maternal characteristics and the effect of a nuchal cord on perinatal outcomes.

	Absence of Nuchal Cord (*n* = 366)	Presence of Nuchal Cord (*n* = 100)	*p*
Maternal age (weeks)	25 (12–46)	25 (15–44)	0.814 ^∫^
Ethnicity			0.376 ^∂^
White	39.4% (142/360)	34.7% (33/95)	
Black	9.2% (33/360)	6.3% (6/95)	
Mixed	51.4% (185/360)	58.9% (56/95)	
BMI (Kg/m^2^)	30.8 (14.4–56.2)	29.4 (20.5–50.9)	0.277 ^∫^
Number of pregnancies	2 (1–10)	2 (1–8)	0.167 ^∫^
Number of deliveries	2 (0–8)	1 (0–6)	0.035 ^∫^
High-risk pregnancy	41.2% (150/364)	36.0% (36/100)	0.347 ^∂^
Gestational age (weeks)	39.1 (37.0–41.6)	39.7 (37.0–41.4)	0.006 ^∫^
Type of delivery			<0.001 ^∂^
Vaginal	60.4% (221/366)	99.0% (99/100)	
Cesarean section	39.6% (145/366)	1.0% (1/100)	
Forceps	0.0% (0/366)	0.0% (0/100)	
Birthweight (grams)	3200 (1120–4370)	3113 (1995–4140)	0.206 ^∫^
Labor duration (minutes)	60.0 (6–679)	30 (8–900)	0.193 ^∫^
Apgar score at the 1st minute	9 (1–10)	8 (2–10)	0.014 ^∫^
Apgar score at the 5th minute	9 (7–10)	9 (6–10)	0.636 ^∫^
Arterial cord blood pH	7.24 (6.8–7.46)	7.27 (6.98–7.44)	0.020 ^∫^
Venous cord blood pH	7.29 (6.91–7.44)	7.30 (6.80–7.43)	0.191 ^∫^

BMI: body mass index; Mann–Whitney ^∫^: median (minimum-maximum); Chi-squared ^∂^: percentage (*n*/*N*). *p* < 0.05.

**Table 2:** Risk of adverse perinatal outcomes according to the presence of a nuchal cord during delivery.

	Absence of Nuchal Cord (*n* = 366)	Presence of Nuchal Cord (*n* = 100)	OR (95% CI)	*p*
Apgar score at the 1st minute < 7	4.9% (18/366)	7.0% (7/100)	1.46 (0.59–3.59)	0.413
Apgar score at the 5th minute < 7	0.0% (0/366)	2.0% (2/100)	18.6 (0.886–391.0)	0.993
Meconium at birth	6.3% (23/366)	12.0% (12/100)	2.03(0.97–4.24)	0.059
Acidemia at birth	7.1% (26/366)	4.0% (4/100)	0.54 (0.18–1.60)	0.262
Oxygen therapy	3.8% (14/366)	3.0% (3/100)	0.77 (0.21–2.76)	0.697
Neonatal intensive care unit admission	2.5% (9/366)	2.0% (2/100)	0.81 (0.17–3.81)	0.789
Neonatal death within 48 h	0.3% (1/366)	0.0% (0/100)	1.21 (0.04–30.0)	0.601
Fetal death at delivery	0.0% (0/366)	0.0% (0/100)		
Adverse composite outcomes	18.0% (66/366)	21.0% (21/100)	1.21 (0.69–2.09)	0.500

OR: odds ratio calculated according to the presence of a nuchal cord; Chi-squared: percentage (*n*/*N*). Binary logistic regression. *p* < 0.05.

**Table 3:** The presence of a loose or tight nuchal cord according to maternal characteristics and the effect of the presence of a loose or tight nuchal cord on perinatal outcomes.

	Loose Nuchal Cord (*n* = 82)	Tight Nuchal Cord (*n* = 18)	*p*
Maternal age (weeks)	25.0 (15–44)	26.0 (16–42)	0.828 ^∫^
Ethnicity			0.576 ^∂^
White	34.6% (27/78)	35.3% (6/17)	
Black	5.1% (4/78)	11.8% (2/17)	
Mixed	60.3% (47/78)	52.9% (9/17)	
BMI (Kg/m^2^)	28.8 (20.5–50.9)	32.9 (23.7–38.6)	0.045 ^∫^
Number of pregnancies	2.0 (1.0–8.0)	2.0 (1.0–4.0)	0.165 ^∫^
Number of deliveries	2.0 (0.0–6.0)	1.0 (0.0–4.0)	0.022 ^∫^
High-risk pregnancy	34.1% (28/82)	44.4% (8/18)	0.410 ^∂^
Gestational age (weeks)	39.7 (37.0–41.4)	39.9 (38.0–41.0)	0.003 ^∫^
Number of cord nuchal	1.0 (1.0–4.0)	1.0 (1.0–2.0)	0.357 ^∫^
Type of delivery			0.638 ^∂^
Vaginal	98.8% (81/82)	100.0% (18/18)	
Cesarean section	1.2% (1/82)	0.0% (0/18)	
Forceps	0.0% (0/82)	0.0% (0/18)	
Birthweight (grams)	3130 (1995–2495)	3090 (2495–3860)	0.082 ^∫^
Labor duration (minutes)	30.0 (6.0–900.0)	40.5 (30.0–146.0)	<0.001 ^∫^
Apgar score at the 1st minute	9.0 (3.0–10.0)	8.0 (2.0–9.0)	0.007 ^∫^
Apgar score at the 5th minute	9.0 (6.0–10.0)	9.0 (6.0–10.0)	0.726 ^∫^
Arterial cord blood pH	7.28 (6.98–7.44)	7.23 (7.07–7.40)	0.025 ^∫^
Venous cord blood pH	7.31 (6.80–7.43)	7.26 (7.06–7.38)	0.099 ^∫^

BMI: body mass index; Mann–Whitney ^∫^: median (minimum-maximum); Chi-squared ^∂^: percentage (*n*/*N*). *p* < 0.05.

**Table 4:** Risk of adverse perinatal outcomes according to the presence of a loose or tight nuchal cord during delivery.

	Loose Nuchal Cord (*n* = 82)	Tight Nuchal Cord (*n* = 18)	OR (95% CI)	*p*
Apgar score at the 1st minute < 7	3.7% (3/82)	22.2% (4/18)	7.52 (1.51–37.31)	0.014
Apgar score at the 5th minute < 7	1.2% (1/82)	5.6% (1/18)	4.76 (0.28–79.99)	0.278
Meconium at birth	12.2% (10/82)	11.1% (2/18)	0.90 (0.18–4.51)	0.898
Acidemia at birth	3.7% (3/82)	5.6% (1/18)	1.54 (0.15–15.81)	0.712
Oxygen therapy	1.2% (1/82)	11.1% (2/18)	10.12 (0.86–118.46)	0.065
Neonatal intensive care unit admission	2.4% (2/82)	0.0% (0/18)	0.87 (0.04–18.90)	0.503
Neonatal death within 48 h	0.0% (0/82)	0.0% (0/18)		
Fetal death at delivery	0.0% (0/82)	0.0% (0/18)		
Adverse composite outcomes	18.3% (15/82)	33.3% (6/18)	2.23 (0.72–6.90)	0.163

OR: odds ratio calculated according to the presence of tight nuchal cord; Chi-squared: percentage (*n*/*N*). Binary logistic regression. *p* < 0.05.

## Data Availability

The data presented in this study are available on request from the corresponding author.
